# Predictors of Malignancy in Thyroid Nodules Classified as Bethesda Category III

**DOI:** 10.3389/fendo.2022.806028

**Published:** 2022-02-16

**Authors:** Xiaoli Liu, Jingjing Wang, Wei Du, Liyuan Dai, Qigen Fang

**Affiliations:** Department of Head Neck and Thyroid, Affiliated Cancer Hospital of Zhengzhou University, Henan Cancer Hospital, Zhengzhou, China

**Keywords:** thyroid disease, papillary thyroid carcinoma (PTC), Bethesda category III, fine needle aspiration, AUS/FLUS

## Abstract

**Objective:**

To determine predictors of malignancy in surgically treated Bethesda category III nodules.

**Methods:**

Patients with surgically treated thyroid nodules classified as Bethesda category III were retrospectively enrolled. The association between clinical/sonographic features and postoperative pathology was evaluated using univariate and multivariate analyses.

**Results:**

A total of 188 nodules from 184 patients were included. The overall malignancy rate was 54.3%. In univariate analysis, male sex, aspect ratio >1, microcalcification, unclear boundary, *BRAF^V600E^* mutation, and nuclear atypia were significantly associated with malignant disease in Bethesda category III nodules. Multivariate analysis confirmed that male sex, aspect ratio >1, microcalcification, and *BRAF^V600E^* mutation were independent predictors of malignant disease.

**Conclusions:**

Malignant disease was common in Bethesda category III nodules, and surgical treatment was strongly indicated in the presence of male sex, aspect ratio>1, microcalcification, and *BRAF^V600E^* mutation.

## Introduction

An increasing number of thyroid nodules are being detected due to the rapid development of high-resolution ultrasound (1). Although most do not require surgical treatment, it is essential to differentiate malignant from benign tumors. Fine needle aspiration (FNA) offers a reliable method for evaluating the nodule’s properties (2, 3). The Bethesda System for Reporting Thyroid Cytopathology has been formulated for standardizing FNA results among different institutions (4) and consists of six categories. Because of the challenges of tumor management, special attention is usually paid to Bethesda III, which is defined as atypia of undetermined significance/follicular lesion of undetermined significance (AUS/FLUS) (5).

The Bethesda System estimates that the malignant risk is less than 15% for the Bethesda III nodules and recommends a repeat FNA (4). However, recurrent AUS/FLUS may still occur in about one-third of the cases (6). More importantly, there is substantial evidence that the real malignant risk of AUS/FLUS is significantly higher than the estimated incidence (7–13). Therefore, it is important to identify reliable indicators, such as the *BRAF^V600E^* mutation status, adverse sonographic features, nuclear and architectural atypia, and circulating cell-free DNA integrity, for determining the likelihood of malignancy and guiding clinical decision-making (7–13),. However, to our knowledge, there is still controversy regarding the predictors of malignancy in Bethesda III group thyroid nodules. Therefore, in the current study, we aimed to determine reliable risk factors for malignancy in AUS/FLUS thyroid nodules.

## Patients and Methods

### Ethical Consideration

Henan Cancer Hospital Institutional Research Committee approved our study, and all patients signed informed consent agreements for medical research before receiving the initial treatment. All methods were performed in accordance with the relevant guidelines and regulations.

### Patient Selection

From January 2018 to August 2021, there was a total of 3001 patients who underwent FNA for 3889 thyroid nodules of which 434 nodules had an AUS/FLUS result. The enrolled patients were subjected to the following inclusion criteria: the disease was primary; cytopathology result was presented as AUS/FLUS; and surgical excision was performed for the punctured nodule. Patients without sufficient demographic and clinicopathologic information were excluded.

### Treatment Principle

In our cancer center, thyroid nodules were initially assessed by ultrasound using the Thyroid imaging reporting and data system. FNA was performed for differentiating between benign and malignant disease. If AUS/FLUS was reported, a decision was made to perform direct surgery or continue observation after carefully considering the sonographic features and the patient’s willingness.

### Sonographic/Pathologic Feature Definition

Unclear boundary, irregular shape, solid structure, hypoechogenicity, microcalcification, and aspect ratio (taller than wide) >1 on transverse section were defined as the classic sonographic features of thyroid cancer (7, 8). Nuclear atypia was considered to be present if there was nuclear enlargement, pale chromatin, inclusion-like appearance and irregular nuclear contours in different degrees. Architectural atypia was considered to be present if there were follicular cells in microfollicular pattern or crowded three-dimensional groups and with scant colloid (2).

### Fine Needle Aspiration Technique

FNA was performed using a 22-gauge needle. Each aspiration was repeated at least three times to ensure sufficient collection. Immediately after aspiration, the sample in the needle was prepared for cytological assessment using liquid-based cytology.

### Statistical Analysis

Association between clinical/sonographic variables and postoperative malignancy (incidental papillary thyroid carcinoma was not included) was first evaluated using the Chi-square test. Subsequently, the significant factors in univariate analysis were analyzed in multivariate analysis to identify the independent predictors of malignancy. All statistical analyses were performed using SPSS 20.0, and a p-value <0.05 was considered significant.

## Results

A total of 184 patients comprising 144 women and 40 men were included in the analysis. The median age was 50 years (range: 19-83 years). A total of 188 nodules were subjected to surgery. All nodules were solid and demonstrated hypoechogenicity. Unclear boundary, irregular shape, microcalcification, and an aspect ratio >1 were noted in 100 (53.2%), 80 (42.6%), 74 (39.4%), and 14 (7.4%) nodules, respectively. The median size of the nodules was 9.5 mm (range: 3.0-71 mm). The *BRAF^V600E^* mutation was identified in 52 (27.7%) nodules. Postoperative pathology revealed benign disease in 86 (45.7%) nodules and malignancy in 102 (54.3%) nodules. In the 102 malignant nodules, four were diagnosed as follicular thyroid carcinoma (FTC), while the rest were diagnosed as papillary thyroid carcinoma (PTC). There was no cases of noninvasive follicular thyroid neoplasm with papillary-like nuclear features. Incidental PTC developed in 18 (9.8%) patients. Architectural and nuclear atypia were noted in 67 (35.6%) and 88 (46.8%) nodules, respectively.

Among the 14 nodules with an aspect ratio >1, 12 were malignant, and the malignancy rate was significantly higher than that in the 174 nodules without this feature (p=0.023). In the 74 nodules with microcalcification, the malignancy rate was 75.7%, which was significantly higher than the malignancy rate (40.4%) in the 114 nodules without microcalcification (p<0.001). Malignant disease was detected in 62 of the 100 nodules with an unclear boundary and 40 of the 88 nodules without an unclear boundary, and the difference was statistically significant (p=0.023).

The rate of malignancy in the 52 nodules with *BRAF^V600E^* mutation was significantly higher than the rate of malignancy in the 136 nodules without *BRAF^V600E^* mutation (98.1% and 37.5%, respectively, p<0.001). In the 52 nodules with *BRAF^V600E^* mutation, 49 nodules were confirmed as PTC, and 2 nodules were confirmed as FTC. In the 136 nodules without *BRAF^V600E^* mutation, 49 nodules were confirmed as PTC, and 2 nodules were confirmed as FTC.

Among the 88 nodules with nuclear atypia, malignant disease was noted in 58 cases, while in the 100 nodules without nuclear atypia, malignant disease was noted in 44 cases, and the difference was statistically significant (p=0.003). There were no apparent associations between other demographic and sonographic features and postoperative malignancy (all p >0.05, **Table 1**).

**Table 1:** Univariate analysis of the association between clinical and sonographic variables and postoperative pathology.

Variable	Postoperative pathology		p
	Benign (n = 86)	Malignant (n = 102)	
Age			
≤50	40	58	
>50	46	44	0.157
Sex			
Male	12	32	
Female	74	70	0.005
Unclear boundary			
Yes	38	62	
No	48	40	0.023
Irregular shape			
Yes	34	46	
No	52	56	0.442
Microcalcification			
Yes	18	56	
No	68	46	<0.001
Aspect ratio>1			
Yes	2	12	
No	84	90	0.023
Size			
≤9.5	40	52	
>9.5	46	50	0.541
BRAFV^600E^ mutation			
Yes	1	51	
No	85	51	<0.001
Architectural atypia			
Yes	33	34	
No	53	68	0.472
Nuclear atypia			
Yes	30	58	
No	56	44	0.003

In further multivariate analysis, the presence of male sex, aspect ratio >1, microcalcification, and *BRAF^V600E^* mutation were independently associated with about a 3.7-fold, 6.7-fold, 5.7-fold, and 6.7-fold increased risk of malignant disease, respectively (**Table 2**).

**Table 2:** Multivariate analysis of the association between clinical and sonographic variables and postoperative pathology.

Variable	p	OR	95%CI
Sex	0.004	3.741	1.536-9.109
Aspect ratio>1	0.027	6.767	1.245-36.781
Microcalcification	<0.001	5.787	2.720-12.303
BRAF^V600E^ mutation	<0.001	6.793	2.830-16.305
Nuclear atypia	0.312	1.425	0.717-2.830

## Discussion

The most important findings of the current study were that more than half of the AUS/FLUS nodules were malignant, and male sex, aspect ratio >1, microcalcification, and *BRAF^V600E^* mutation were the independent predictors of malignancy. Surgical treatment is recommended for an AUS/FLUS nodule if any of these features were present.

The malignancy rate in AUS/FLUS nodules varies from 17% to 83.3%, according to the current evidence (10, 14). Although the rate in the present study was also within this range, it was surprising to note there is great variability in the incidence. In a study by Zhao et al. (10), the authors attributed their high incidence rate to selection bias and strict cytological criteria for malignancy diagnosis. In our cancer center, rather than being based on molecular findings, a decision of surgical treatment was usually made if the clinical/radiological examination revealed malignancy. On the other hand, a misdiagnosis of thyroid cancer is not considered acceptable in China because of the poor doctor-patient relationship. Consequently, cytopathologists have tended to adopt more stringent diagnostic criteria to diagnose malignancy, which has caused an underdiagnosis of thyroid nodules of other categories by FNA. Additionally, the decision of surgical treatment for AUS/FLUS nodules was usually made after considering the patient’s wishes. The psychological endurance of patients appears to differ widely according to their cultural background (15).

A significant relationship between the male sex and malignant disease has never been described for AUS/FLUS nodules. Consequently, the current study might be the first to show that the male sex is associated with an additional 2.7-fold risk of malignant disease. Liu et al. (16) analyzed the risk factors for central lymph node metastasis in cN0 PTC and found that the male sex was an independent predictor with an odds ratio (OR) of 5.6. Recently, Zuhur et al. (17) confirmed the prognostic significance of male sex in differentiated thyroid carcinoma and reported that according to the American Thyroid Association low-risk category, male sex predicted a markedly shorter disease-free survival. The induction of worse biologic behaviors and prognosis of the male sex might have also partially contributed to our finding. Moreover, although men only accounted for 21.7% of the total cases, those who had more adverse sonographic features had been screened meticulously by the surgical team.

Sonographic features are the most frequently analyzed predictors for AUS/FLUS nodules. Huang et al. (18) retrospectively presented the results of 272 patients with surgically treated Bethesda category III nodules and revealed that both microcalcification and shape were independent malignant risk factors. Gao et al. (19) reviewed 14 studies including 2,405 AUS/FLUS nodules to highlight the predictive value of adverse sonographic characteristics, which consisted of hypoechogenicity, irregular margin, micro/macro/disrupted rim calcification, taller-than-wide shape, increasing size during follow-up, and increased vascularization. The authors reported that in the presence of any one of these suspicious features, ultrasound had a pooled sensitivity and specificity of 0.75 and 0.48, respectively, and with any two or three suspicious features, the sensitivity and specificity increased to 0.77 and 0.71, respectively. Alshahrani et al. (20) noted that nodules with multiple numbers, irregular margins, microcalcification, and hypoechogenicity were significantly more likely to be malignant based on an analysis of 187 patients. A similar finding was also confirmed by Cho et al. (21), Erdogan-Durmus et al. (2), and in the present study. This suggests that highly suspicious sonographic features could be helpful and reliable for clinical decision-making.

Nuclear atypia has been widely analyzed for its reliability in predicting malignancy in AUS/FLUS nodules. Lim et al. (13) examined 137 Bethesda III nodules, and malignant lesions were noted in 27.0% of the cases. When the nodules were classified into two subgroups based on the presence of nuclear atypia, the malignancy rate was significantly higher in nodules showing nuclear atypia (36.8% vs. 14.7%, p <0.01). A similar finding was also described by Guleria et al. (22) and Mosca et al. (23). However, nuclear atypia was one of the three atypia patterns that included architectural atypia and the Hurthle cell type described by Xu et al. (14). They noted that nodules with nuclear atypia had the highest malignancy rate among the three groups, although it was not statistically significant. Kaymaz et al. (24) reviewed the pathologic results of 209 AUS/FLUS nodules and found that the overall rate of malignancy was 27.8%, which decreased to 26.4% if only the nodules with nuclear atypia were assessed. This suggests that all nuclear properties are not equally effective in predicting the malignancy risk. Our study also failed to establish an association between nuclear atypia and malignant disease. Additional studies are needed to clarify the question.

The *BRAF^V600E^* mutation is the most common genetic alteration in thyroid cancer, it could even occur in 82.0% of T1a PTCs (25). Suh et al. (9) reported that the *BRAF^V600E^* mutation was the second most influential independent predictor of malignancy in AUS/FLUS nodules and if combined with the Korean Thyroid Imaging Reporting and Data System, it could reduce unnecessary surgeries. Our study also confirmed the reliability of the *BRAF^V600E^* mutation in predicting malignancy.

Limitations of the current study must be acknowledged. Firstly, since it was a retrospective study, there was an inherent bias. Secondly, our sample size was not large enough; hence, future studies with a larger sample size need to be conducted.

In summary, malignant disease is common in AUS/FLUS nodules, and surgical treatment is strongly indicated in the presence of male sex, aspect ratio >1, microcalcification, and *BRAF^V600^* ^E^ mutation.

## Data Availability Statement

The original contributions presented in the study are included in the article/supplementary material. Further inquiries can be directed to the corresponding author.

## Ethics Statement

Henan Cancer Hospital Institutional Research Committee approved our study, and all patients signed informed consent agreements for medical research before receiving the initial treatment.

## Author Contributions

All the authors made the contribution in study design, manuscript writing, studies selecting, data analysis, study quality evaluating, and manuscript revising. All authors have read and approved the final manuscript.

## Conflict of Interest

The authors declare that the research was conducted in the absence of any commercial or financial relationships that could be construed as a potential conflict of interest.

## Publisher’s Note

All claims expressed in this article are solely those of the authors and do not necessarily represent those of their affiliated organizations, or those of the publisher, the editors and the reviewers. Any product that may be evaluated in this article, or claim that may be made by its manufacturer, is not guaranteed or endorsed by the publisher.
